# A cluster randomized controlled trial to test the feasibility and preliminary effectiveness of a family dementia caregiver intervention in Vietnam: The REACH VN study protocol

**DOI:** 10.1097/MD.0000000000012553

**Published:** 2018-10-19

**Authors:** Tuan Anh Nguyen, Huong Nguyen, Thang Pham, Trong Hung Nguyen, Ladson Hinton

**Affiliations:** a Quality Use of Medicines and Pharmacy Research Centre, School of Pharmacy and Medical Sciences, University of South Australia, North Terrace, Adelaide, SA, Australia; b Hamilton College, University of South Carolina, Columbia, South Carolina; c National Geriatric Hospital, Number 1A Phuong Mai, Dong Da, Ha Noi, Viet Nam; d Department of Psychiatry and Behavioral Sciences, University of California Davis, Sacramento, California.

**Keywords:** cluster randomized controlled trial, dementia, family caregiver intervention, Resources for Enhancing All Caregivers Health in Vietnam, Vietnam

## Abstract

**Background:**

Resources for Enhancing All Caregivers Health in the Department of Veterans Affairs (REACH VA) is an evidence-based intervention supporting family dementia caregivers that has been shown to improve caregiver outcomes for culturally diverse populations in the United States. However, this model has not been tested in low- and middle-income countries (LMICs) including Vietnam, where community-based psychosocial interventions are urgently needed. The objectives of this study are to assess the feasibility and preliminary effectiveness of a culturally adapted version of the Resources for Enhancing All Caregivers Health in Vietnam (REACH VN).

**Methods:**

A cluster randomized controlled trial (RCT) will be conducted over a 6-month period in Soc Son district located in Hanoi. An expected sample of 10 to 12 communes, representing approximately 50 dementia primary caregivers, will be randomized to either the REACH VN intervention or an enhanced control condition. Inclusion criteria for the caregiver include age ≥18, family member who provides the most day-to-day care for person with dementia, and a total score for the brief (4 item) Zarit Burden Scale of ≥6. Over the course of 2 to 3 months, each participant in the intervention group will receive the REACH VN intervention comprised of 4 core sessions on problem solving, mood management/cognitive restructuring, stress management, and communication, and up to 2 additional sessions based on caregiver's needs. The enhanced control group will receive a single session that provides verbal and written information on dementia. Caregiver outcomes will be assessed at baseline (i.e., time of enrolment) and 3 months. The feasibility will be assessed with regard to recruitment, retention, treatment adherence, treatment fidelity, and assessment processes. For preliminary effectiveness, we will examine caregiver burden as the primary outcome and changes in caregiver depressive symptoms and in Alzheimer disease knowledge as secondary outcomes.

**Discussion:**

This is the first study to test community-based family dementia caregiver intervention in Vietnam. Results from this study will provide the foundation for a larger effectiveness trial and broader dissemination in Vietnam and may help inform efforts to develop similar community-based family dementia caregiver support programs in other LMICs.

**Trial registration:**

ClinicalTrials.gov, ID: NCT03587974. Published online July 16, 2018

## 1 Background

In the next several decades, because of a dramatic demographic transition, Vietnam and other low- and middle-income countries (LMICs) will experience a substantial increase in the number of elderly people, including those with dementia. The proportion of the population aged ≥60 years in Vietnam was 10% in 2015 and is predicted to rise to 28% by 2050.^[1]^ Based on current estimates of 4% to 8% of Vietnamese aged ≥60 years having dementia,^[2,3]^ Vietnam will have >2 million people living with dementia by the year 2050.

Dementia and cognitive impairment are costly and among the leading causes of disability and dependency in old people.^[4,5]^ The total cost for dementia in 2018 is estimated to be 1 trillion US dollars worldwide and it will double by 2030.^[4]^ The impact of dementia is not only on patients, but also on their families, communities, and society.^[4]^ The families of people with dementia have to spend significant amount of time, effort, and money to support and care for patients with dementia. The high costs of long-term care for people with dementia also strains health and social systems, as well as government budgets.^[5]^ Therefore, there is an urgent need for LMICs, including Vietnam to develop a comprehensive national plan and health care initiatives to address dementia as a public health issue and to develop effective approaches to both prevent the disease and to provide adequate support to family caring for persons with dementia in the community.

A range of interventions have been developed to respond to dementia, including both pharmacological and non-pharmacological therapies.^[6]^ Research has shown that non-pharmacological approaches (e.g., strengthening capacity of family caregivers of people living with dementia via education, skill-building, and stress reduction) are effective in improving caregiver's outcomes and have lower risks and adverse reactions than pharmacological treatments, and result in a better health outcomes for patients and caregivers.^[7–10]^

Among these approaches, Resources for Enhancing All Caregivers Health in the Department of Veterans Affairs (REACH VA) is an evidence-based model supporting dementia caregivers with many advantages. REACH and the follow-up REACH II study, the intervention models upon which REACH VA is based, have been tested and disseminated in a number of countries.^[10]^ Their effectiveness has been demonstrated not only in multicultural countries such as the United States, but also in Hong Kong—a country with similar cultural characteristics as in Vietnam.^[10,11]^ Compared with the original REACH and REACH II interventions, REACH VA is briefer, easier to apply, and potentially more sustainable.^[11–13]^ It has also been shown to be as effective in improving outcomes for family caregivers compared with previous versions of the REACH intervention.^[11,14]^

The REACH interventions, including REACH VA, have not yet been tested in LMICs including Vietnam, where community-based approaches are urgently needed. Despite many promising advantages, it is crucial for a new, multi-component psychosocial intervention like REACH VA to be adapted to the local context of Vietnam (e.g., culture and resources) and tested before being implemented nationally.^[15]^ Wainberg et al^[16]^ developed a model for adapting evidence-based behavioral interventions to a new culture. The model consists of 4 phases namely: selecting an existing model through literature review and discussing with stakeholders; conducting a formative phase to guide the development of the manual; piloting to refine the treatment; and evaluating the adapted treatment using a randomized controlled trial (RCT) design.

Following this model, we have conducted a literature review and chosen the REACH VA model after discussion with collaborators in Vietnam, conducted formative interviews with both family caregivers, community leaders, and health workers to modify the REACH VA intervention, developed adapted Vietnamese versions of the caregivers material and interventionist guide, and conducted a case series to pilot the intervention. This study protocol describes the design of a cluster RCT to evaluate our adapted REACH VA intervention (REACH VN). The primary objective of this study is to examine the feasibility of REACH VN in Vietnam. A secondary objective is to conduct exploratory analyses to examine the preliminary effectiveness of REACH VN.

## 2 Methods

### 2.1 Study design

A two-arm, 3-month follow-up, cluster RCT will be conducted. A cluster RCT design was chosen because of concerns about “contamination effects” if the intervention and control subjects were drawn from the same geographic area (i.e., commune). The communes in Vietnam are small and people in a commune often know each other well. If subjects from the intervention groups talk to the controlled group about the intervention, it would contaminate the study. Clusters (i.e., communes) will be randomized into group A (REACH VN) or group B (enhanced usual care). All eligible dementia caregiver-care recipient dyads in the intervention communes (group A) will receive the REACH VN intervention while those in the enhanced control communes (group B) will receive enhanced usual care, enhanced by education and receipt of written information on Alzheimer disease and related dementia. At the conclusion of the study, all participants in the enhanced control group will be offered an optional caregiving training workshop.

### 2.2 Study setting

Soc Son district in Hanoi, Vietnam, a community that has worked closely with the National Geriatric Hospital of Vietnam on other projects, has been selected as the site for this study. The National Geriatric Hospital has maintained an existing registry of people with dementia in Soc Son and people with dementia from this registry and their family caregivers will be screened and invited to participate.

### 2.3 Eligibility criteria

Inclusion criteria are family caregivers of persons diagnosed with dementia (Clinical Dementia Rating—CDR score of 1.0 or above^[17]^) living in one of the designated clusters (communes). In addition, to be eligible, the primary family caregivers must be 18 years of age or older, the family members who is most involved in dementia patients’ day-to-day care, and have a total score for the brief (4 items) Zarit Burden Scale of 6 or higher.^[18]^ Caregivers with significant cognitive impairment will be excluded from the study.

### 2.4 Description of the REACH VN and enhanced control conditions

Participants in the intervention group will receive a culturally adapted intervention that based on the REACH VA intervention.^[11]^ While a number of adaptations were made to the REACH VA model (paper in preparation), including translation of manuals and materials into Vietnamese, the REACH VN is very similar in most respects. The intervention itself consists of 4 “core” training sessions on problem solving, mood management/cognitive restructuring, stress management (e.g., signal breath, mindfulness medication, pleasant event scheduling), and communication, plus up to 2 additional sessions based on caregiver's needs and clinical judgment^[11,12,19]^ and will be delivered over the course of 2 to 3 months.

Each session will last approximately 1-hour and will be delivered by an interventionist certified to deliver the intervention. The interventionists will be healthcare professionals and include nurses, physicians, and social workers. The sessions will be administered in the home (or other setting of the subject's choice such as a clinic) and include several components, including psychoeducation, stress reduction, skill-building. While it is anticipated that most sessions will be face-to-face, the interventionist will also have the option of conducting sessions by phone. The intervention will be tailored to the needs and preferences of the caregiver. At the first session, each participant will receive written materials on dementia as well as a caregiver notebook with detailed information on a variety of topics related to dementia caregiving and will serve as a reference during the intervention. After each session, an observer will complete the Treatment Delivery Form to document the delivery of key components of the intervention.

The enhanced control condition will consist of a single face-to-face session that will occur at the time of enrollment and be held in the caregiver's home (or another place of their choosing). Caregivers will be educated about the nature of dementia and provided with written educational materials. In addition, any safety issues identified during the visit will be addressed by research staff. All enhanced control participants will be invited to attend a caregiving training workshop that will be held after the conclusion of the study.

### 2.5 Outcomes measures

The primary objective of this study is to assess feasibility of the intervention. The feasibility of the study method will be assessed by using accepted guidelines^[20]^ with respect to recruitment, retention, treatment adherence, treatment fidelity, and assessment processes. Recruitment feasibility will be judged by the number of family caregivers screened in order to identify caregivers meeting eligibility criteria and who consent to participation. We will record the subject retention in both arms of the study and track intervention adherence. As part of the feasibility assessment, we will assess treatment fidelity (i.e., interventionist adherence to the treatment protocol) through both direct observation of sessions by one of the study leaders and by having an independent observer assess delivery of key components of the intervention at the end of each session. A comparison between the content actually delivered in sessions (reviewing interventionist notes) and the treatment manual's checklist will also be performed. In addition, the feasibility of the study administration will be measured by the percentage of baseline and 3-month assessments completed, timeliness and length of assessments, and completeness of collected data.

While the study will not be powered to examine efficacy, we will conduct analyses to examine caregiver outcomes. For the exploratory analyses of intervention effectiveness, we will use a 4-item version of the Zarit Burden Scale as the primary outcome for caregivers.^[18]^ Secondary caregiver outcomes for this analysis will include change in depressive symptoms as measured by the Patient Health Questionnaire 4 (PHQ-4)^[21]^ and change in Alzheimer disease knowledge.

### 2.6 Sample size and study duration

This cluster RCT is a pilot study, exploring the preliminary efficacy of the REACH VN intervention and the feasibility of the intervention and study methods to further refine the intervention and prepare for a larger RCT to fully test the intervention effectiveness. Therefore, it is not powered optimally to examine the effectiveness. An expected sample of 10 to 12 communes consisting of approximately 50 dementia primary caregivers will be enrolled into the trial over a 6-month period. For participants, the intervention will consist of 4 to 6 sessions delivered over 2 to 3 months.

### 2.7 Randomization process

Designated communes will be randomly allocated to either the intervention or the enhanced control group with a 1:1 allocation. Randomization will be conducted by one of the principal investigators (PIs) who is not familiar with the characteristics of the communes being randomized. All eligible dementia caregiver-care recipient dyads living in the intervention communes will be the intervention participants while those living in the control communes will be control participants.

### 2.8 Subject recruitment and reimbursement

Participants for this study will be drawn from a registry of individuals in Soc Son who have been previously diagnosed with dementia by neurologists from the National Geriatric Hospital (NGH) in collaboration with local health clinics. NGH staff will contact families of elders with dementia who reside in the designated geographic areas (i.e., communes), introduce the study, and screen caregivers for eligibility criteria. Caregivers who meet inclusion criteria will be asked for permission to have research staff contact to set up an initial visit (i.e., enrollment visit). During the enrollment visit, the research staff will provide additional information for the study and complete verbal consent. Participants in both the REACH VN intervention and the enhanced control group will be reimbursed the equivalent of US $9 for each session they participate in. As part of the consent process, all caregivers are informed that they can withdraw at any time.

### 2.9 Blinding

Because randomization will occur at the level of individual clusters, individual participants will not be blind to the condition they will receive. Staff conducting baseline assessments will not be blind to participant allocation. Staff conducting outcome assessments at 3 months will be blind to allocation.

### 2.10 Assessment of outcomes

Study outcomes will be assessed at the time of enrollment and at 3-months after their enrollment visit. The baseline survey and 3-month follow-up assessments will take approximately 30 to 45 minutes.

### 2.11 Statistical analyses

Data will be analyzed using Stata 15 (StataCorp, College Station, TX)^[22]^ or a similar statistical software to generate all statistical reports. Data will be analyzed on an intention to treat basis. For continuous data, the normality of data distribution will be tested using the Shapiro-Wilk test. Mean and standard deviation (SD) will be presented for normally distributed data while median and interquartile rage (IQR) will be used otherwise. For categorical data, frequencies and percentages will be presented. To evaluate efficacy, a general linear model, analogous to an ANCOVA model, which includes the baseline assessment as a covariate, will be used to compare the groups; this model will further incorporate a cluster random effect to account for the clustered design of the trial.

### 2.12 Data monitoring, safety, and quality control

The Institutional Review Boards (IRBs) or similar committee at UC Davis, the University of South Carolina and the NGH will review the study protocol. The IRBs will maintain ongoing oversight of the risks and benefits of the study and ensure compliance with institutional and NIH guidelines. The PI will be responsible for notifying the IRBs at each institution of adverse events in a timely fashion or any changes to study protocol.

All study staff will be instructed in the rules of confidentiality and data safeguarding for the study. Patient-identifiable information will be removed from all data. Participant baseline and follow-up data and medical chart abstraction data will be directly entered into a secure, password-protected database by study staff, and will only be linked to a unique study identification number. All participant contact information will be maintained in locked file cabinets at the NGH or the UC Davis Department of Psychiatry. The identity of study participants will be known by the study staff only as needed for purposes of the study. Periodic reports will be generated using graphs and tables summarizing the status of participant, recruitment, and data collection. Missing data will be routinely rectified. Raw data will be kept for 1 year following the publication of the final reports on the study.

This study will have a safety officer (SO) but will not have a data safety monitoring board (DSMB). The rationale for not having a DSMB is that this is a single-site, minimal risk clinical trial with a relatively small number of subjects in total. The lead investigators will meet quarterly with SO during the clinical trial. During these meetings, the SO responsibility will be to review the progress of the study, adverse events, procedures for maintaining the confidentiality of data, and the quality of data collection, management, and any analyses. The SO will also review any issues that arise in terms of conflict of interest.

The data safety-monitoring plan helps to ensure the safety of participants, the validity of data, and the appropriate termination of the study if significant benefits or risks are uncovered or if it appears that the trial cannot be concluded successfully. The PI and research team will be responsible for monitoring data safety and integrity. Specific data safety and monitoring tasks include:

(1) Monitoring patient accrual: Informed consent and patient enrollment processes will be routinely monitored. Data will be collected on the numbers of study eligible participants; those completing informed consent; those refusing study participation; and those unable to be located. Periodic reports will be generated with this information and reviewed by the PI and study staff. When indicated, appropriate action will be initiated to address accrual problems (e.g., incorrect telephone numbers, methods of approaching patients).
(2) Monitoring severe adverse events: All study staff will be trained in procedures to identify and handle adverse events. All adverse events will also be reviewed by the study SO. If an adverse event is determined to be likely due to study participation, the PI will report the conclusions of the review to study investigators and to the IRBs of UC Davis, the University of South Carolina, the National Geriatric Hospital, and NIH within 1 week. Adverse events determined to be unlikely to be related to study participation will be reported to the IRBs and NIH as part of routine progress reports.
(3) Monitoring study progress: Caregiver outcome data will be collected by study staff and monitored by the project manager and, if necessary, by the biostatistician. The PI and project manager will meet regularly to monitor recruitment, attrition, patient safety, and study implementation; the entire study team.

## 3 DISCUSSION

This is a unique study because it is the first to test a culturally-adapted and evidence-based community family dementia caregiver intervention in Vietnam. If successful, the intervention will be an important step towards a larger study to test its effectiveness in Vietnam. The study team is interdisciplinary, including American, Australian, and Vietnamese specialists who are not only experts in different fields relating to dementia care such as gerontology, psychiatry, neurology, pharmacy, social work and nursing, but also has extensive field work experience in Vietnam. The team members from the United States and Australia, 2 of whom are bilingual in English and Vietnamese, have deep understanding of Vietnamese culture as well as the health care system. The Vietnamese study team is based at the NGH, a leading institution in Vietnam for national geriatric policy, as well as geriatric training, healthcare, and research. The collaboration with a leading institution in Vietnam will enhance the future wider “scaling-up” of the final intervention model in Vietnam and the translation of this research into policies to enhance sustainability.

This study fills an important and acute gap in Vietnam. While there are a number of psychosocial family caregiver interventions conducted in Asia over the past 2 decades,^[10,23–27]^ the vast majority have been conducted in high income countries with very few in LMIC. The intervention which will be used in this research is an evidence-based model that has been systematically adapted to ensure that it will align with the local culture, language, and resources. We believe that our project has the potential to significantly influence dementia care practice in Vietnam and REACH VN and might eventually be a model suitable for adoption and use in other LMICs.

## Acknowledgments

Danielle Harvey PhD provided consultation on statistics sections of this manuscript. Jennifer Martingdale-Adams PhD and Linda Nichols PhD provided consultation on adaptation of the intervention.

## Author contributions

All authors contributed to the paper's conception and design. Tuan Anh Nguyen and Ladson Hinton contributed to the writing of the manuscript. Huong Nguyen and Thang Pham reviewed and commented on the manuscript. All authors read and approved the final manuscript.

**Conceptualization:** Tuan Anh Nguyen, Huong Nguyen, Thang Pham, Trong Hung Nguyen, Ladson Hinton.

**Funding acquisition:** Huong Nguyen, Thang Pham, Trong Hung Nguyen, Ladson Hinton.

**Investigation:** Tuan Anh Nguyen, Huong Nguyen, Thang Pham, Trong Hung Nguyen, Ladson Hinton.

**Methodology:** Tuan Anh Nguyen, Huong Nguyen, Thang Pham, Trong Hung Nguyen, Ladson Hinton.

**Writing – original draft:** Tuan Anh Nguyen.

**Writing – review & editing:** Huong Nguyen, Thang Pham, Ladson Hinton.

Tuan Anh Nguyen orcid: 0000-0002-9528-9278
